# A new diagnostic strategy which uses a luminol-H_2_O_2_ system to detect helminth eggs in fecal sediments processed by the Helmintex method

**DOI:** 10.1371/journal.pntd.0008500

**Published:** 2020-07-30

**Authors:** Vivian Favero, Carolina De Marco Veríssimo, Angela R. Piovesan, Alessandra L. Morassutti, André A. Souto, Hélio R. Bittencourt, Vanessa F. Pascoal, Catieli G. Lindholz, Malcolm K. Jones, Renata P. Souza, Francine De Vargas Rigo, Célia R. Carlini, Carlos Graeff-Teixeira

**Affiliations:** 1 Research Group on Biomedical Parasitology, School of health and life sciences, Pontifícia Universidade Católica do Rio Grande do Sul, Porto Alegre, Brazil; 2 Neurotoxins Laboratory (LaNeuroTox), School of Medicine, Brain Institute and School of Medicine, Pontifícia Universidade Católica do Rio Grande do Sul, Porto Alegre, Brazil; 3 Centre for One Health and Ryan Institute, School of Natural Sciences, National University of Ireland Galway, Galway, Ireland; 4 Graduate Program in Cellular and Molecular Biology, Center of Biotechnology, Universidade Federal do Rio Grande do Sul, Porto Alegre, Brazil; 5 Polytechnic School, Pontifícia Universidade Católica do Rio Grande do Sul, Porto Alegre, Brazil; 6 School of Veterinary Science, The University of Queensland, Brisbane, Qld, Australia, 4072; 7 Infectious Diseases Unit, Center for Health Sciences, Federal University of Espirito Santo, Vitoria, Brazil; Wellcome Sanger Institute, UNITED KINGDOM

## Abstract

Schistosomiasis remains a serious public health problem in tropical regions, affecting more than 250 million people. Sensitive diagnostic methods represent key tools for disease elimination, in particular in areas with low endemicity. Advances in the use of luminol-based chemiluminescent techniques have enabled greater sensitivity and speed in obtaining results in different diagnostic settings. In this study, we developed a luminol-H_2_O_2_ chemiluminescence (CL) method to detect *Schistosoma mansoni* eggs in human fecal sediments processed by the Helmintex (HTX) method. After *S*. *mansoni* eggs were incubated with a solution of luminol-H_2_O_2_ the light emission was detected and measured by spectrophotometry at 431 nm for 5 min, using detection and counts of eggs by bright field optical microscopy as a reference. CL intensity was found to correlate with different sources and numbers of eggs. Furthermore, our results showed that the CL method can distinguish positive from negative samples with 100% sensitivity and 71% specificity. To our knowledge, this is the first study to report the use of CL for the diagnosis of helminths from fecal samples. The combination of the HTX method with CL represents an important advance in providing a reference method with the highest standards of sensitivity.

## Author summary

Schistosomiasis, the infection caused by parasites of the genus *Schistosoma*, is still a significant health problem in many countries, despite some successful control efforts. In areas where parasite load is low and few eggs are released in feces, most diagnostic tests fail to detect the infections. Helmintex is a very sensitive, but time consuming, egg-detection method, with most time being consumed in observations of samples at the microscope. Here we report standardization of an innovative luminol-H_2_O_2_ chemiluminescence reaction triggered by *Schistosoma mansoni* eggs that may improve their detection in feces. Improved diagnostic tests are urgently required as part of the schistosomiasis elimination efforts.

## Introduction

Among the parasitic diseases that occur in developing countries, schistosomiasis is the second most important in terms of socioeconomic effects, prevalence, and public health, second only to malaria [1–4]. More than 250 million individuals worldwide are infected with schistosomes, of which three species, *Schistosoma haematobium*, *Schistosoma mansoni*, and *Schistosoma japonicum*, are responsible for most infections [3, 5–7]. Led by the World Health Organization, many endemic countries are engaged in efforts to eliminate schistosomiasis [7].

Highly sensitive diagnostic tests are crucial for detecting the parasite in late stages of elimination and for confirming that transmission has been interrupted [8]. Ideally, a suitable diagnostic method should be sensitive, inexpensive, easy to perform and rapid. Considerable efforts have been focused on molecular tools that meet these requirements [8–11]. At the same time, a direct antigen test that detects a *S*. *mansoni* antigen, the circulating cathodic antigen (CCA) has been used extensively for detection of schistosomiasis in point of care field situations (POC-CCA) [11–14]. This method has also exhibited better performance than the long-standard diagnostic, the Kato-Katz fecal egg smear [15] in localities with high endemicity [16–20]. However, the POC-CCA antigen test is much less accurate in endemic foci where low intensity infections predominate [2, 21, 22].

While most direct egg-detection diagnostic methods, such as Kato-Katz, exhibit low sensitivity while demonstrating high specificity, one recent innovation excels in both measures. The Helmintex (HTX) diagnostic method enables the direct isolation of *S*. *mansoni* eggs from clinical samples for subsequent microscopic examination because of the strong binding between eggs and paramagnetic particles (PMP). HTX exhibits 100% sensitivity for egg numbers greater than 1.34 eggs per gram of feces [23] and is recommended as a gold (or reference) standard for evaluating the diagnostic accuracy of other methods [23]. Despite the exceptionally high sensitivity of the HTX method, aided by recent improvements, introduced by Favero and collaborators [24], the method remains laborious. The rate-limiting aspect of the HTX method is the final step, in which the final sediment produced after affinity purification of eggs is examined in a compound microscope.

During development of the HTX method, three types of PMP, differing only in their surface functionalization, were tested for binding efficiency. The most efficient PMP, in terms of proportions of eggs recovered in seeding experiments, were those coated with streptavidin [23], and, thus, it was hypothesized that biotin-like molecules are present on the surface of *S*. *mansoni* eggs. This hypothesis was later confirmed, thereby leading to the successful application of a chemiluminescence (CL) reaction to more rapidly detect eggs present in sediment processed by the HTX method. Therefore, here we report the development and standardization of an innovative luminol-H_2_O_2_ system for the rapid detection of *S*. *mansoni* eggs in human fecal sediments processed by the HTX method.

## Methodology

### Ethics Statement

The *Schistosoma mansoni* life cycle is maintained in the laboratory in Swiss mice and *Biomphalaria glabrata* snails (Minas Gerais, Brazil) [25]. Mice were infected percutaneously with 150 cercariae released by the snails. This research was approved by the Animal Ethics Committee of PUCRS (Approval No. PUCRS CEUA 15/00443) under Law n° 11.794 of October 2008.

Work with human samples was approved by the Ethics Committee of the René Rachou Research Center (FIOCRUZ, 21824513.9.0000.5091) and the Research Ethics Committee of PUCRS (48809715.1.0000.5336). Human fecal samples were obtained from epidemiological studies performed in the municipalities of Januária, Minas Gerais (15° 29’ 44”S, 44° 21’ 45”W), and Estância, Sergipe (29° 38’ 57”S, 51° 10’ 25”W), Brazil [21, 26]. All volunteers provided informed written consent.

### Isolation of *Schistosoma mansoni* eggs and miracidia from infected mice

Parasite eggs were obtained from experimentally infected mice and from feces of naturally-infected humans. Notations for the different sources of eggs are provided in Table 1. For this study, 50 days after mice were infected with *S*. *mansoni*, they were anesthetized with 5% isoflurane (BioChimico, Brazil) and then received an intraperitoneal injection of 100 μL sodium heparin (5000 IU/mL, HEPAMAX-S, Brazil). After 10 min, the animals were euthanized with CO_2_ anesthetic depression. *S*. *mansoni* adult worms were subsequently recovered by perfusion and livers were removed. Blood samples as positive control (M-blood) for CL reactions, as luminol is used in forensics to identify blood.

**Table 1:** List of samples subjected to luminol-H_2_O_2_ chemiluminescence reactions according to their biological material and host.

Sample label	Biological Material	Host
**L-Eggs**	Digestion of liver tissue according to Dalton and colleagues (25)	Mouse
**Iv-Eggs**	*In vitro* cultured worms	Mouse
**HF-eggs**	Fecal samples from naturally infected individuals, HTX method,–PMP	Human
**HF-Eggs-PMP**	Fecal samples from naturally infected individuals, HTX method, + PMP	Human
**Neg-HF**	Fecal samples from healthy, non-infected individuals, submitted to HTX method	Human

PMP: paramagnetic particles; HTX: Helmintex.

Liver eggs (L-eggs)—Mouse livers were removed for subsequent isolation of eggs following Dalton and colleagues [27]. In brief, L-eggs were obtained by digesting the livers with 10 μg collagenase B (Sigma-Aldrich, Germany) and 10 μg penicillin-streptomycin (Gibco, USA) in 1X PBS, at 37°C overnight. The resulting tissue material was then sieved through a 150 μm mesh and the eggs that were recovered were further purified by sedimentation and centrifugation in a Percoll gradient (Sigma-Aldrich, Germany).

Miracidia—An aliquot of the purified L-eggs was added to distilled water and exposed to light in a chamber, at 27°C for 1 h to allow hatching of the miracidia.

*In vitro* eggs (Iv-eggs)—were obtained from adult *S*. *mansoni* worms cultured in DMEM medium (Gibco, USA) supplemented with glucose (4.5 g/L), fetal bovine serum (10% v/v) (CRIPION, Brazil), and 10% penicillin-streptomycin at 37°C and 5% CO_2_. The medium was changed daily and laid eggs were collected.

*Schistosoma mansoni* eggs (L-eggs and IV-eggs) and miracidia were successively isolated from other debris with repeated (4 times) capture by pipetting in PBS under an optical microscope. L-eggs, Iv-eggs and miracidia were separated into groups of 10, 25, and 50 in tubes containing 1X PBS (pH 7.0) and were stored at −20°C.

### Isolation of *Schstosoma mansoni* and *Ascaris lumbricoides* eggs from human samples

Fecal samples were obtained from humans from previous field surveys in Januária, Minas Gerais, and Estancia, Sergipe (see Section 2.1), Brazil. Fecal samples from participants confirmed in those surveys to be infected with *S*. *mansoni*, were used as sources of eggs for CL experimentation. These were separated into two groups, according to the method used to process them, as described in Table 1.

Human fecal eggs (HF-eggs): These samples were subjected to the HTX method [24, but without addition of PMP in the final sediment.

Human fecal eggs with PMP (HF-eggs-PMP)- Fecal samples were subjected to the HTX method with PMP added to the final sediment [24].

*Ascaris lumbricoides* eggs*-* Fecal samples were subjected to the HTX method with PMP. In some samples with heavy *A*. *lumbricoides* infections, the eggs of that nematode were also present in the final sediment, although they are not bound by PMP.

All egg and shell isolates from human samples (HF-eggs, HF-eggs-PMP), empty eggshells, and *A*. *lumbricoides* eggs, were successively isolated from fecal debris and parasite material with repeated capture and washing in PBS (4 times) by pipette under an optical microscope. The isolated samples were separated into groups of 10, 25, and 50 in tubes containing 1X PBS (pH 7.0) and stored at −20°C.

### Processing of human negative control samples

**Fig 1:**
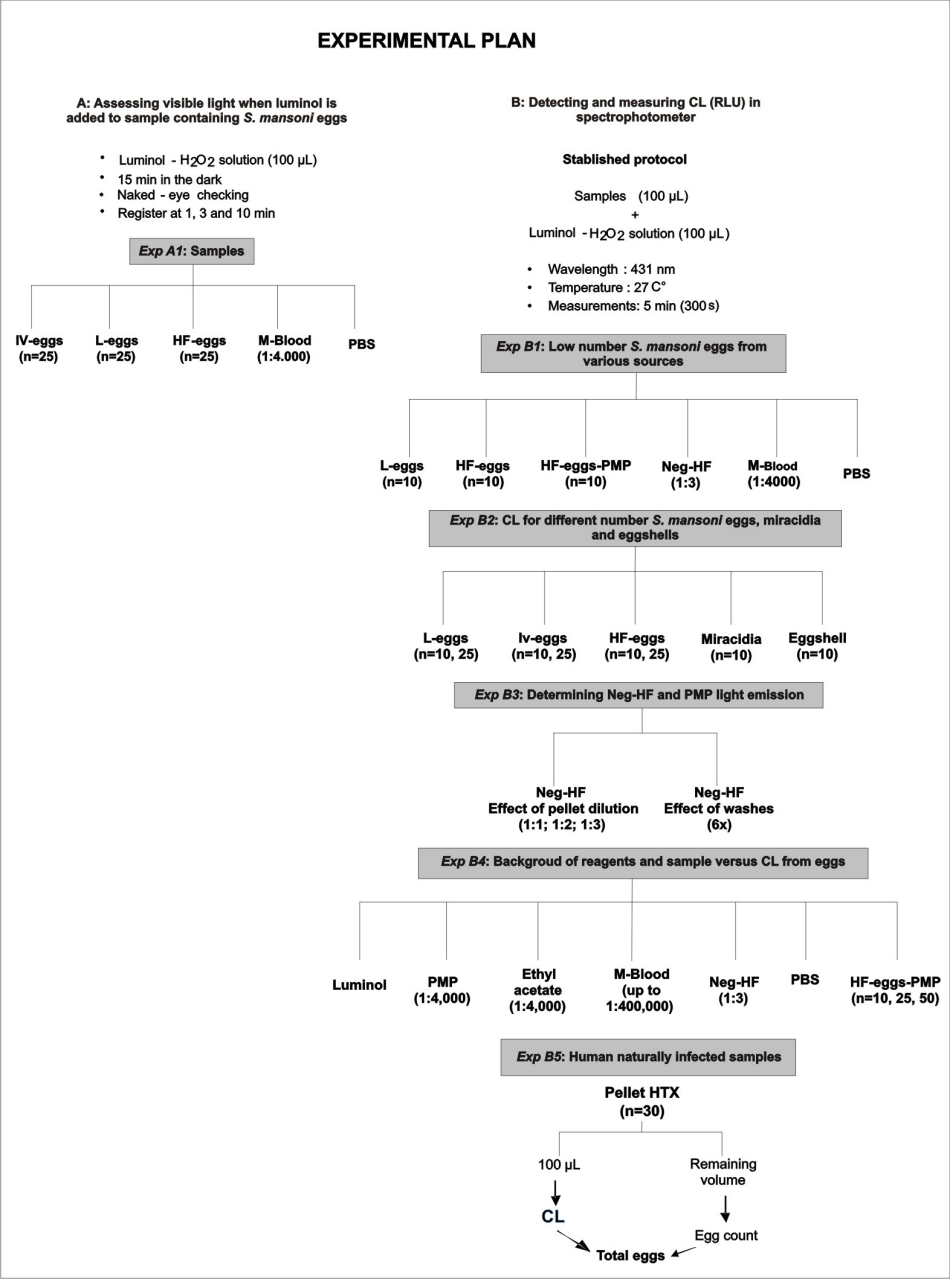
Schema showing the experimental plan used in the current study.

To obtain human negative control samples, stool samples were collected from ten individuals from regions not endemic for schistosomiasis. The samples were processed according to the HTX method [24] and were shown to be negative for eggs after microscopy. The final sediments were pooled, homogenized, and split into two 1 mL aliquots (labeled aliquots 1 and 2) and mixed with 1 mL PBS and 19 μL PMP. After 30 min of orbital agitation, the samples were placed in a magnetic field to isolate a pellet (see section 2.5. Helmintex method). Each pellet obtained from aliquots 1 and 2 were resuspended in PBS (1:1 w/v), washed 6x by centrifugation, 2 min at 200 × g, and suspended in PBS (1:1 w/v). At each of the six wash steps for Aliquot 1, a 100 μL volume was set aside for CL experiments. The pellet obtained from aliquot 2 was diluted 1:1, 1:2, and 1:3 (w/v) in 1X PBS and these samples were stored at -20°C (Fig 1, section 2.7, *Exp*. *B3*).

### Helmintex method

HTX method was performed according to Favero et al. [24]. Briefly, 30 g of feces were fixed in Tween 20 + Ethanol Solution (final concentration of 5% Tween 20 + 35% Ethanol). After fixation and homogenization, the pellet was sieved with a 500 μm metal mesh, transferred to a conical beaker and washed until a clear supernatant was obtained. The resulting sediment was again sequentially sieved through metal meshes with openings of 150 μm and 45 μm, the latter to retain *S*. *mansoni* eggs. The fraction retained by the last sieve (45 μm) was suspended in a 30% (v/v) aqueous ethyl acetate, homogenized and centrifuged for 10 minutes at 200 × g. After discarding the supernatant, the pellet were transferred to a microtube containing 19 μL of PMP (Bangs Labs, USA). The microtubes were allowed to homogenize for 30 min with orbital rotation, and then placed on a magnetic rack (Bangs Labs, USA) for 3 min. Unbound material was discarded before each tube was removed from the rack. The sediment in microtubes were then suspended in 100 μL of 0.9% aqueous NaCl solution (v/v) and stored at -4°C until analyzed. For microscopy analysis, each sediment was suspended and stained in 3% ninhydrin (Sigma-Aldrich, USA) in 70% ethanol (v/v). Each suspension was evenly spread over 5 cm × 2.5 cm filter papers (24 μm pore) (UNIFIL, Brazil), labeled for subsequent identification, and kept for examination by optical microscopy (magnification, 100×).

### Luminol—H_2_O_2_ solution

The luminol-H_2_O_2_ solution used was composed of: 40 mM luminol (Sigma-Aldrich, USA), 10 mM KOH (Nuclear, Brazil), and 17 mM H_2_O_2_ (Sigma-Aldrich, Germany). The solution was always prepared immediately before use and it was protected from light.

### Chemiluminescence experiments

A: Initial verification of visible light emission by naked-eye in samples containing *S*. *mansoni* eggs

*Exp A1*: When activated, luminol emits light in the visible range and is observable as a pale blue light. To test whether schistosome eggs can induce luminol-CL, three independent samples of 25 eggs from the L-eggs, Iv-eggs, HF-eggs samples were incubated with luminol-H_2_O_2_ solution. A sample containing only M-blood diluted in 1X PBS (1:4,000) was used as positive control, while a 1X PBS only volume was used as negative control (Fig 1). All samples were made to 100 μL in wells of 96-well opaque polystyrene plates (Thermo Scientific, EUA), to which 100 μL luminol-H_2_O_2_ solution was added to each well. The plates were incubated at 27°C for 15 min in a darkroom. The samples were inspected for luminescence at 1, 3, 10 mins. Samples were imaged using a Nikon Coolpix P600 light.

B: Detection and measurement of chemiluminescence in the samples by spectrophotometer

Once egg-induced chemiluminescence was confirmed as described above, all subsequent experiments were analyzed by spectrophotometry using a Molecular Devices, SpectraMax M3 spectrophotometer. All experiments were conducted at 27°C. The optimum wavelength was determined by scanning CL-positive samples at wavelengths ranging from 300 to 600 nm. Blue luminescence observed with the naked-eye corresponds to wavelengths ranging from 380 nm to 495 nm, the expected range for luminol-induced luminescence. A 431 nm CL peak was detected in all of the samples. From these experiments, the optimum spectrophotometry conditions were obtained and used in all subsequent experiments: Detection wavelength: 431 nm; temperature: 27°C, incubation time in luminol-H_2_O_2_ solution: 5 minutes (300 seconds). The CL values were expressed in relative light units (RLU) per second (s), an indirect estimation of light intensity (431nm), since spectrometry does not directly measure photons, but electromagnetic radiation [28].

*Exp B1*- *Assessing CL from low numbers of S*. *mansoni eggs obtained from different sources*: The light emitted after luminol exposure was assessed in independent triplicate samples, each containing 10 eggs of L-eggs, HF-eggs-PMP or HF-eggs, made up to 100 μL volumes. As controls, samples of 100 μL Neg-HF diluted 1:3 in PBS, 100 μL M-blood diluted 1:4,000 in PBS (v/v), and 100 μL of PBS (Fig 1).

*Exp B2- Assessing CL from low and intermediate numbers of S*. *mansoni eggs*, *miracidia and eggshells*: CL measurements were collected for six independent replicates of 10 and 25 eggs from samples of L-eggs, Iv-eggs, and HF-eggs. To verify the light emitted by miracidia and eggshells, six independent replicates of 10 miracidia and 10 eggs shells were analyzed under the same conditions (Fig 1).

*Exp B3- Determining the CL from eggs negative fecal human samples*: To better discriminate infected from non-infected fecal samples and to optimize handling of the final HTX processed sediments, we tested whether washing of samples in PBS has an effect on any background luminescence induced by fecal samples. For this we measured CL of Neg-HF after it had been processed through 6 centrifugation and resuspension steps as described for Aliquot 1 of Neg-HF in section 2.4 above, or in 1:1, 1:2 and 1:3 dilutions of Neg-HF(Aliquot 2). All the samples made to 100 μL volume, to which luminol solution (100 μL) was added (Fig 1).

*Exp B4 –Assessing background CL induced by reagents in the HTX method*: Six independent replicates of 100 μL luminol solution (tested for auto-chemiluminescence), 100 μL PMP diluted 1:4,000in 1X PBS, 100 μL ethyl acetate diluted 1:4,000 in 1X PBS (v/v), 100 μL M-blood diluted at 1:400, 1:4,000, and 1:400,000 in 1X PBS, 100 μL Neg-HF diluted 1:3 in PBS, 1X PBS, and samples of 10, 25, and 50 HF-eggs-PMP were analyzed following the protocol described above. In addition, six independent replicates of samples containing 10, 25, and 50 *A*. *lumbricoides* eggs were evaluated (Fig 1).

*Exp B5 –Assessing the correlation between CL and presence of S*. *mansoni eggs in human fecal samples from endemic regions for schistosomiasis*: Human stool samples (n = 30) from patients from Januária and Estância, Brazil, were processed according to the HTX method [24]. From the final sediments, 100 μL of each was set aside for CL testing, whereas the remaining sediments were subjected to standard HTX analysis to quantify the egg burden of *S*. *mansoni* eggs (expressed as eggs per gram of feces, epg) for comparative enumeration of the numbers of eggs present in the sediments for CL. Then, the 100 μL aliquots submitted to spectrophotometry were transferred by pipette to a microscope slide for counting of eggs by optical microscopy (Fig 1).

### Statistical analysis

The six replicates of each experiment generated series of measurements from 0–15 min (t0–t15) which are presented in two-dimensional graphs of CL intensity (RLU-Relative Light Units) versus time (s). The graphs present the mean CL intensity for the groups, although area under the curve (AUC) values were calculated individually to allow comparisons to be made. The latter were calculated based on the sum of the area of the rectangle with base of 19 seconds, the interval between measurements, and the height being the midpoint between f(t) and f(t+15), being “t” the time in seconds, and 15 seconds the end of observation period. The final AUC value is the sum of all of the rectangle areas. To reduce the magnitude of the values, the sums were logarithmized. After the AUC values were compared, a receiver operating characteristic (ROC) curve analysis was performed to establish a cut-off value to provide discrimination of positive samples. Comparisons among the different groups were performed by using the Kruskal-Wallis test; the correlations were performed by the Spearman test, both followed by a multiple comparisons nonparametric test, with a level of significance of 0.05. Box plots were constructed to present these results. The statistical software program, SPSS 17.0, was used to perform the statistical analyses for this study.

## Results

### Suspensions containing *Schistosoma mansoni* eggs emit a strong and persistent visible light when exposed to luminol-H_2_O_2_ solution

Visible blue light could be observed by naked eye and recorded by camera when 25 *S*. *mansoni* eggs from L-eggs, Iv-eggs, and HF-eggs samples were incubated with a luminol-H_2_O_2_ solution (Fig 1, *Exp A1*). In particular, the L-eggs and Iv-eggs preparations, both obtained from mouse infections, induced CL for 10 min, while the HF-eggs only induced light emission for 3 min. Similarly, the positive control (M-blood diluted 1:4,000) exhibited an intense emission of light for only 3 min, while the PBS-only negative controls did not induce luminol-H_2_O_2_ CL. From these data, we infer that immature eggs (Iv-eggs) and samples of hepatic eggs (L-eggs) from mice emit visible blue light strongly for at least 10 minutes, whereas eggs from human samples (HF-eggs) emit visible blue light for somewhere between 3 minutes after exposure to Luminol-H_2_O_2_.

### Light intensity measured by spectrophotometry is proportional to the number of eggs in a sample

**Fig 2:**
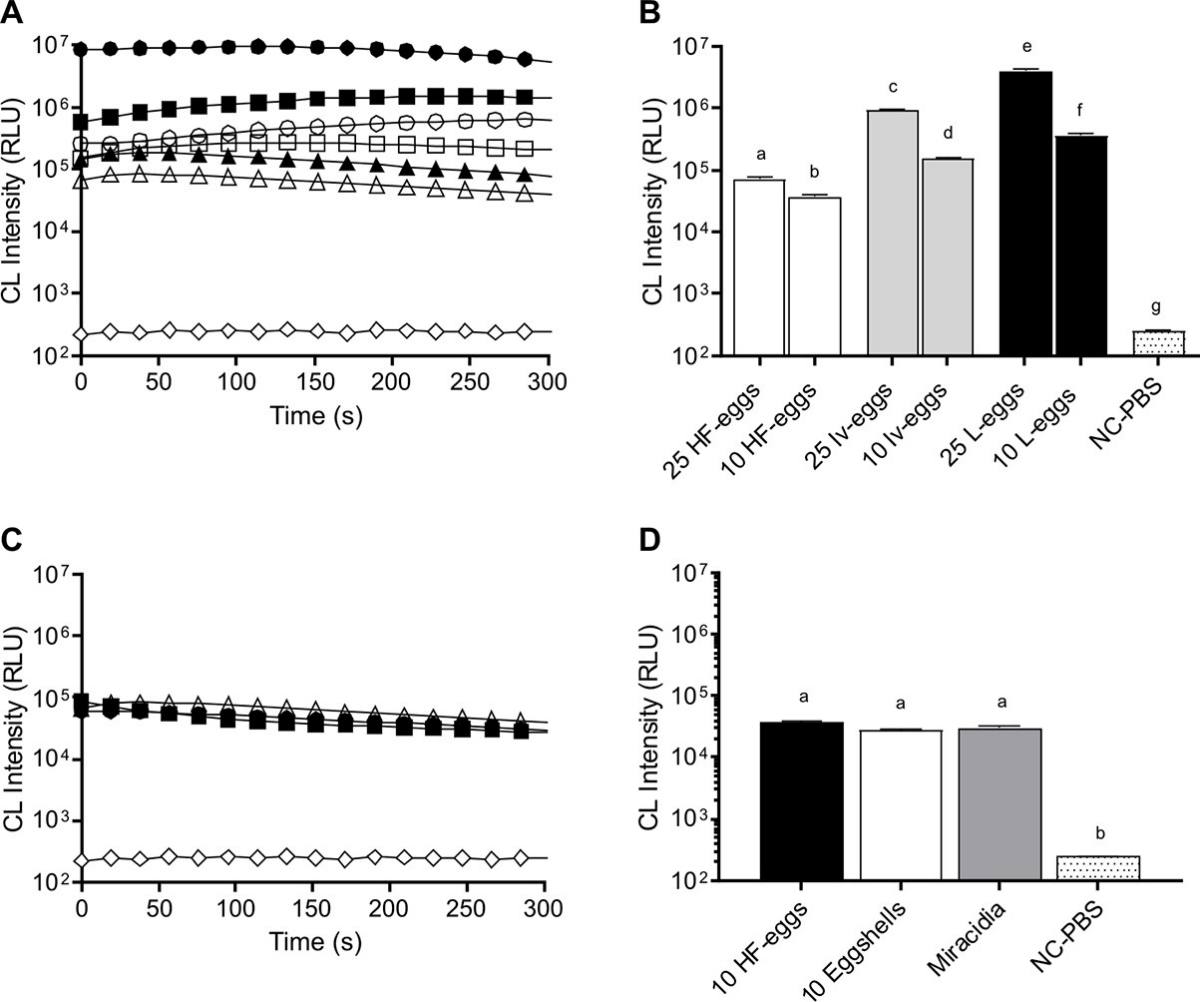
Quantification of CL in *Schistosoma mansoni* eggs samples. CL intensity profile (in relative light units, “RLU”) obtained at 431 nm with a spectrophotometer: **A**-10 (white circle, white square, white triangle) and 25 (black circle, black square, black triangle): L-Eggs (white circle, black circle), Iv-eggs (white square, black square), HF-eggs (white triangle, black triangle) and Negative Control (NC-PBS) containing PBS 1x (white lozenge). **B**-Bar graph representing the estimated AUC values of 25 egg versus 10 egg (HF-eggs, L-eggs, and Iv-eggs) samples. **C**- CL intensity profile for eggshells (black circle), miracidia (black square), HF-eggs (white triangle) and Negative Control (NC-PBS) containing PBS 1x (white lozenge). **D**- Bar graph representing the estimated AUC values for samples containing HF-eggs, eggshells, and miracidia as indicated. Significant differences were not observed. Bar graphs represent AUC values for CL intensity plotted with time. Different letter labels indicate significant differences (*p*≤0.05), and similar letter labels indicate non-significant differences, according to the multiple comparison test.

CL generated by different numbers of *S*. *mansoni* eggs was quantitatively analyzed at 431 nm by spectrophotometry (Fig 1, *Exp B2*). The CL generated by L-eggs, Iv-eggs, and HF-eggs preparations varied according to the number of eggs present in the samples (Fig 2A). Furthermore, a statistical analysis to compare AUC values for CL generated by each sample showed that those containing 25 eggs emitted significantly greater CL intensity than those containing 10 eggs (Fig 2B). There was no significant difference in light intensity generated by samples containing 10 HF-eggs, 10 miracidia, and 10 eggshells (Fig 2C and 2D).

### *Schistosoma mansoni* eggs isolated with the HTX method can be distinguished from controls based on CL intensity

**Fig 3:**
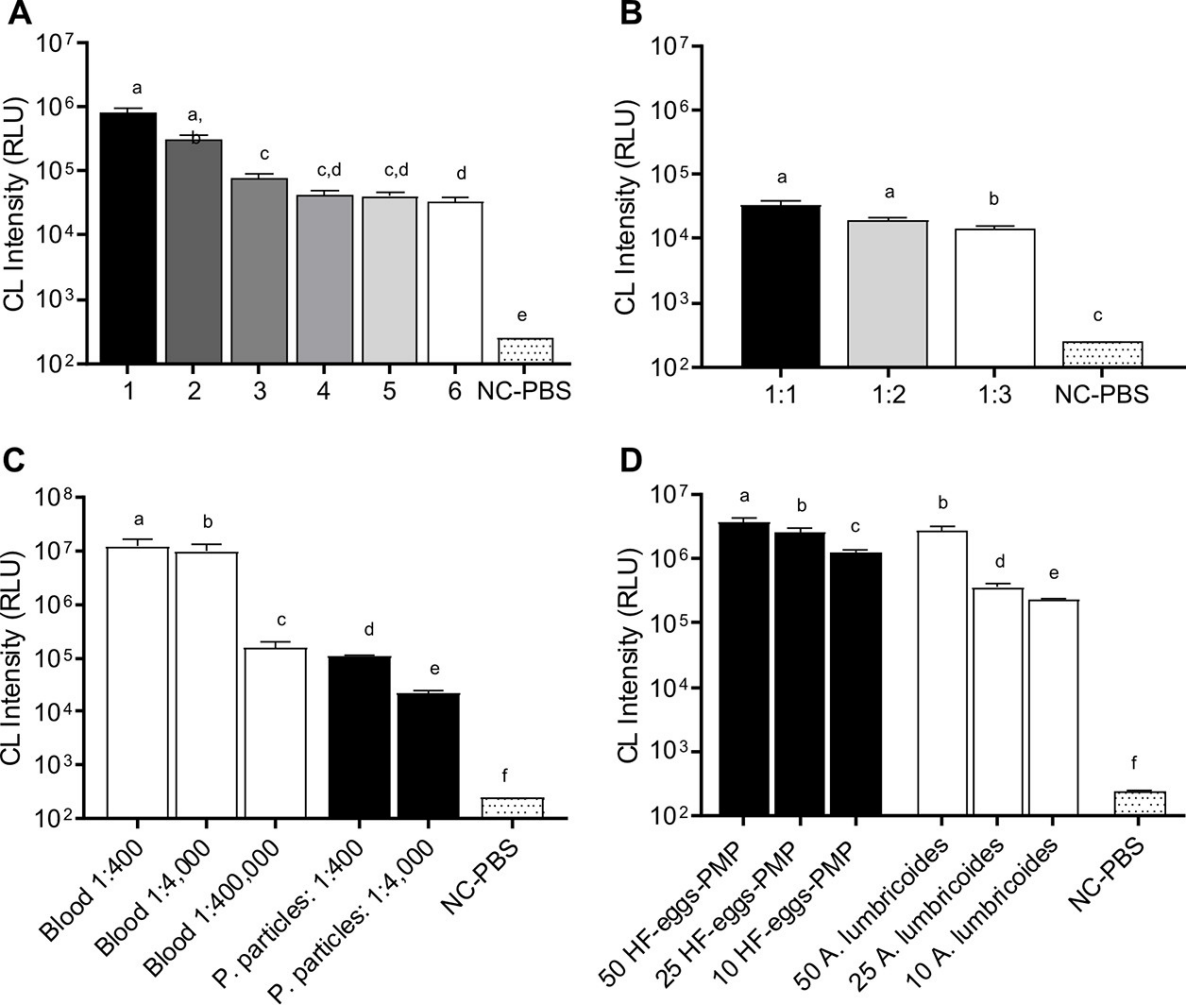
Comparison of CL signals between *Schistosoma mansoni* samples and potential sources of false-positivity. Bar graphs represent AUC values for CL intensity plotted on logarithmic scale**. A**- CL intensity of fecal sediment produced by HTX, from non-infected human individuals (Neg-HF). The numbers on the X-axis represent the numbers of washes to which the samples were subjected to prior to incubation with luminol-H_2_0_2,_ showing that CL intensity is reduced with each wash. **B**-CL intensity of fecal sediment produced by HTX, from non-infected human individuals (Neg-HF). In this experiment, Neg-HF samples were diluted 1:1, 1:2, and 1:3 in PBS and the CL was significantly reduced (*p*≤0.05) after dilution 1:3 in PBS. **C**- CL intensity profile for whole mouse blood diluted to 1:400, 1:4,000, and 1:400,000, PMP diluted to 1:400 and 1:4,000 and Negative Control (NC-PBS) containing PBS 1x. **D**- CL of 50, 25, and 10 HF-eggs-PMP, which were isolated from feces from naturally infected human being compared to the CL generated by 50, 25, and 10 eggs of *A*. *lumbricoides*. Bar graphs represent AUC values for CL intensity plotted with time. Different letter labels indicate significant differences (*p*≤0.05), and similar letter labels indicate non-significant differences, according to the multiple comparison test.

The M-blood and Neg-HF (1:3) samples generated strong CL signals when incubated with the luminol-H_2_O_2_ solution (Fig 1, *Exp B3* and *B4*), suggesting that fecal blood and fecal material generally can produce background CL. When the M-blood samples were diluted, and with increased washes or dilution of Neg-HF samples (Fig 3A, 3B and 3C), the CL intensity of both blood and negative fecal samples decreased markedly (Fig 3A and 3B). In both cases, CL intensity diminished markedly with decreasing concentration (dilution), a scenario that could be expected in any fecal sample processed by HTX, which includes multiple washing steps.

In schistosomiasis-endemic regions, humans can also be subject to polyparasitism and carry infections of soil-transmitted and food-borne helminths. As a representative species, encountered CL induced by *A*. *lumbricoides* was assessed. Aliquots containing 10, 25, or 50 eggs of *S*. *mansoni* or *A*. *lumbricoides* isolated from human fecal samples in the field were assessed for luminol-CL. The samples containing 10 or 25 *A*. *lumbricoides* eggs induced CL that was less intense than those containing equal numbers of *S*. *mansoni* eggs (HF-eggs-PMP) (Fig 3D). However, the samples containing 50 *A*. *lumbricoide*s eggs exhibited the same light intensity as the *S*. *mansoni* samples containing 25 or 50 eggs (Fig 3 D).

**Fig 4:**
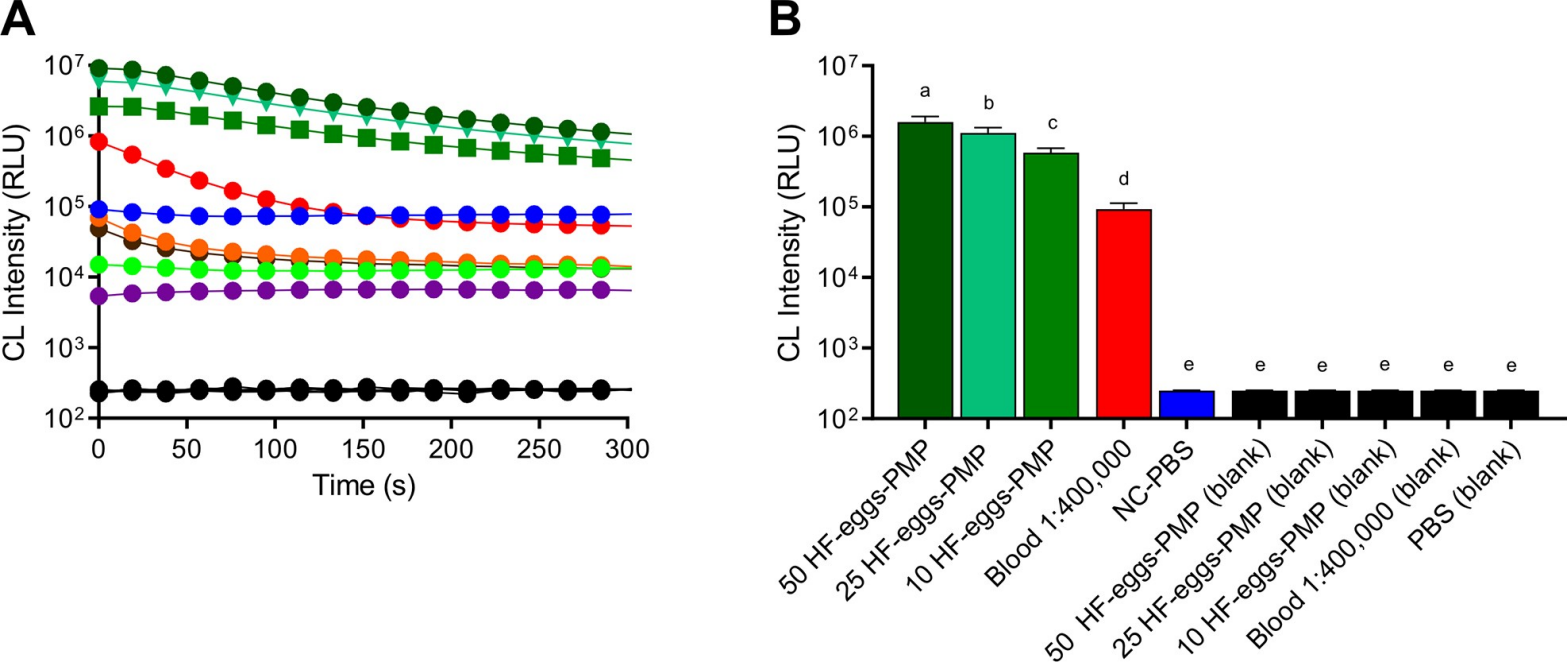
Differential CL intensities generated by *Schistosoma mansoni* eggs and reagents used during fecal samples processing. **A**- CL intensity profile at 431 nm of 50 (forest green circle), 25 (spring green triangle) and 10 (green square) *S*. *mansoni* before eggs obtained from HF-eggs-PMP. Mouse blood (M-blood) diluted 1:400,000 as a positive control (red circle). Negative fecal sediment, (Neg-HF,brown circle) and reagents used in the HTX method, including: ethyl acetate (blue circle), paramagnetic particles (PMP, orange circle), PBS (lime green circle) and luminol (purple circle). All of the “blank” samples, without adding luminol solution, converged to the same CL intensity (black circle). **B**- Bar graphs represent AUC values for CL intensity plotted with time. Different letter labels indicate significant differences (*p*≤0.05), and similar letter labels indicate non-significant differences, according to the multiple comparison test.

Low CL intensity was also observed when several chemical reagents from the HTX method were individually tested (Fig 4). When luminol was absent (blank samples), significantly lower CL values were obtained (Fig 4).

### Fecal samples from *Schistosoma mansoni*-infected humans can be distinguished from uninfected samples by Luminol-H_2_O_2_ chemiluminescence

**Fig 5:**
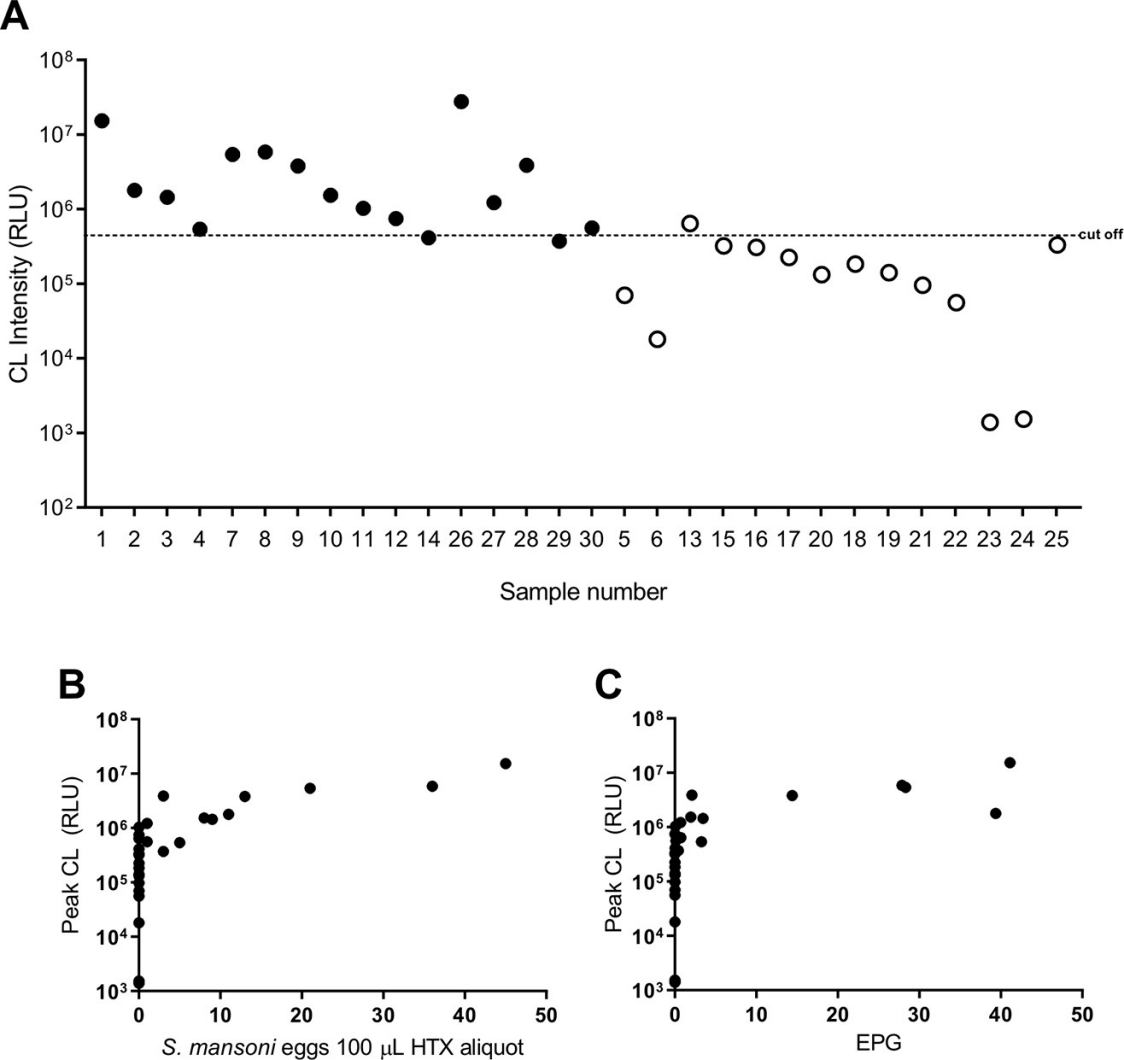
Helmintex associated to CL detection distinguishes *S*. *mansoni* infected and uninfected human samples. **A**- CL peak (RLU) detected from each of the 30 human fecal samples processed by HTX. The horizontal dotted line marks the calculated cut-off value of 3.51 x 10^5^. The black circles represent samples confirmed positive by microscopy and the black triangles samples for which no *S*. *mansoni* eggs were found. All the samples with RLU equal or superior to the cut-off were considered positive in HTX+CL method; **B-** Correlation with 95% confidence interval (Nonparametric correlation–Spearman) between peak CL and number of eggs present in the 100 μL HTX aliquot (R: 0.81, *p*≤0.0001); **C**- Correlation with 95% confidence interval (Nonparametric correlation–Spearman) between CL peak and EPG determined by HTX method) (R: 0.89, *p*≤0.0001).

When HTX-processed samples of human feces were exposed to Luminol-H_2_O_2_ labeling (Fig 1, *Exp B5*), the peak CL obtained from positive human samples varied from 2.95 x 10^7^ to 3.7 x 10^5^ while negative samples varied from 3.3 x 10^5^ to 1.3 x 10^3^ (Fig 5A and Table 2). After the spectrophotometry assays were completed, the same 100 μL sediment aliquots used in this analysis were subjected to examination by optical microscopy for egg counts, following the standard HTX method. Any eggs detected in the aliquot were counted, and also added to the total eggs found in the respective remaining sediment for which egg counts were obtained by standard HTX methodology.

**Table 2:** Correlation of CL peaks and numbers of *Schistosoma mansoni* eggs detected in HTX final sediment from naturally infected human individuals.

Sample number	HTX final sediment volume (μL)	*S*. *mansoni* egg in 100 μL aliquot of HTX sediment analyzed by CL^1^	CL peak (RLU)	N°. eggs in HTX sediment	Total *S*. *mansoni* eggs in the sample^2^	EPG^3^
**1**	700	45	1.53×10^7^	1.189	1234	41.13
**2**	1000	11	1.78×10^6^	1.170	1181	39,37
**3**	600	9	1.44×10^6^	95	104	3.47
**4**	400	5	5.40×10^5^	93	98	3.27
**5**	850	0	6.98×10^4^	0	0	0.00
**6**	1500	0	1.80×10^4^	0	0	0.00
**7**	460	21	5.40×10^6^	829	850	28.33
**8**	1700	36	5.85 × 10^6^	800	836	27.87
**9**	630	13	3.80×10^6^	424	432	14.40
**10**	720	8	1.53×10^6^	51	59	1.97
**11**	750	0	1.03 × 10^6^	2	2	0.07
**12**	470	0	7.46×10^5^	0	0	0.00
**13**	760	0	6.42×10^5^	22	22	0.73
**14**	730	0	4.13×10^5^	1	1	0.03
**15**	1540	0	3.21×10^5^	0	0	0.00
**16**	820	0	3.07×10^5^	0	0	0.00
**17**	670	0	2.25×10^5^	0	0	0.00
**18**	580	0	1.84×10^5^	0	0	0.00
**19**	430	0	1.41×10^5^	0	0	0.00
**20**	380	0	1.32×10^5^	0	0	0.00
**21**	1280	0	9.75×10^4^	0	0	0.00
**22**	290	0	5.59×10^4^	0	0	0.00
**23**	740	0	1.39×10^3^	0	0	0.00
**24**	750	0	1.53×10^3^	0	0	0.00
**25**	410	0	3.32×10^5^	0	0	0.00
**26**	1530	4	2.95×10^7^	50	54	1.80
**27**	1220	1	1.22×10^6^	20	21	0.70
**28**	750	3	3.89×10^6^	60	63	2.10
**29**	750	3	3.70×10^5^	10	13	0.43
**30**	380	1	5.60×10^5^	3	4	0.13

HTX: Helmintex; CL: chemiluminescence

1: There is a positive correlation between the values of the column and the CL peak (RLU).

R = 0.81 (*p*< 0.0001).

2: There is a positive correlation between the values of the column and the CL peak (RLU).

R = 0.88 (*p*< 0.0001).

3: There is a positive correlation between the values of the column and the CL peak (RLU).

R = 0.88 (*p*< 0.0001).

We found that light intensity, RLU, correlates with the number of eggs present in the 100 μL HTX aliquot (R: 0.81, *p≤*0.0001), and was also positively correlated with total *S*. *mansoni* eggs in the whole sediment sample and (R: 0.89, *p*≤0.0001) (Fig 5B, 5C and Table 2). Furthermore, a ROC curve analysis was used to establish a cut-off value from which fecal samples tested can be considered positive for *S*. *mansoni* eggs. The cut-off value determined was 3.51 x 10^5^ RLU. This shows that any fecal sample processed by the HTX method and exposed to luminol-H_2_O_2_ solution that emitted light above the cut-off value can be considered positive for *S*. *mansoni* eggs. By applying this cut-off, from CL results obtained for the 30 human samples used in *Exp B5*, and considering that we have the eggs counts for each of sample, we estimated that the HTX-CL test has 100% sensitivity and 71% specificity for the detection of true-positive samples (Fig 5A).

## Discussion

Detection methods based on luminol-CL have exhibited high sensitivity, while also being cost effective [29, 30], factors justifying the use CL system since the 1960’s in the fields of molecular biology and analytical chemistry [31–37]. Luminol-CL arises when molecules with a high oxidation capacity react with luminol resulting in light generation. Among the molecules that generate luminol-CL are peroxidases, as well as molecules containing ferric or ferrous ions, or other divalent ions such as cobalt, copper, and manganese. Other molecules with this oxidative capacity that induce luminol-CL are those containing active redox protein groups (e.g., heme peroxidases, catalase, cytochromes and non-heme biomolecules, such as the iron sulfur enzyme and the electron transfer proteins, rubredoxins and ferredoxins) [38,39].

Previously, proteomic and high resolution scanning electron microscopy have shown that the schistosome eggshell and miracidia contain divalent metal ions such as iron and copper [40–42], most likely incorporated in proteins such as thioredoxin-peroxidase, thioredoxin, superoxide dismutase, and iron-dependent peroxidase [25, 41–43]. It is, therefore, reasonable to postulate that CL emitted by *S*. *mansoni* eggs when exposed to the luminol-H_2_O_2_ solution results from the reaction with these ions contained in eggshells and miracidia. A recent proteomic analysis of schistosome eggs lists thioredoxin, superoxide dismutase, peroxiredoxin and ferritin as components of schistosome eggs. Further, those authors showed significant variation in expression levels of these molecules in immature (isolated from worms in culture) and mature eggs (isolated from faeces) [25]. Such variation in the bio-physical properties observed in the eggs at different developmental stages might explain differences in longevity of CL generated after incubation of luminol-H_2_O_2_ with eggs isolated from *in vitro* cultures (Iv-eggs), from livers (L-eggs), or human faeces (HF-eggs) (Fig 1). This, in turn, highlights the importance of considering possible biological differences between eggs isolated from tissues or faeces, and even between eggs from different hosts and the importance of examining eggs isolated from human samples in studies of schistosome eggs.

The HTX method incorporates washes, sedimentation, filtration and affinity purification of eggs to produce a diagnostic tool with one of the highest sensitivities obtained from among the array of diagnostics available [24]. Recent surveys, conducted in Brazil, show conclusively that HTX outperformed the much-used CCA direct antigen test [21, 26]. However, according to WHO, the practicalities of diagnostics, notably in endemic areas of developing countries require that diagnostics meet the ASSURED criteria, which is they must be affordable, sensitive, specific, user-friendly, rapid and robust, equipment-free, and deliverable [44]. The HTX achieves most of the requirements. Addition of a step that expedites egg detection will enhance the attractiveness of HTX, which, although supremely sensitive and specific, is slowed because of the need for systematic microscopy of samples.

Here we have shown that luminol-H_2_O_2_ exposure of schistosome eggs induces a distinctive and sustained CL that can be distinguished from non-infected samples and backgrounds. Moreover, the intensity of light emitted by eggs isolated from clinical samples, was proportional to the number of eggs in the samples. Most importantly, associating HTX-CL to process clinical samples resulted in the successful distinction of the egg-negative controls from samples positive for *S*. *mansoni*., with 100% sensitivity and 71% especificity. CL intensities similar to those obtained with *S*.*mansoni* eggs were detected when a large (50) number of *A*.*lumbricoides* eggs were assayed (Fig 3D). Improved concentration procedures to solve this limitation of the method are discussed below.

HTX-CL represents a significant improvement of the detection step of HTX, streamlining the method even more than previous modifications implemented by Favero et. al [24]. Our (see Fig 5A) results show that CL peak intensity correlates with eggs per gram, and with the total number of eggs in a sample or in a homogenous aliquot. Accordingly, to use CL result in significantly less time spent screening sediment samples, making the HTX method more cost-effective. Furthermore, of 30 human clinical samples we tested here (Table 2), we could state confidently that 13 samples that showed chemiluminecence above 3.51 x 10^5^ were positive for schistosome eggs, while by HTX and microscopy, 16 samples were egg-positive. Based on this analysis, any clinical sample that emitted CL below the threshold of 3.51 x 10^5^ could be regarded as egg negative, leaving only those samples with CL above the threshold to be investigated further for egg positivity. Omission of the need to screen low-CL samples would result in a time saving of at least 5 hours compared with standard HTX analysis for the same samples, based on the average time for screening a sample of ninhydrin-stained eggs prepared by HTX [24].

Other advantages of HTX-CL include the low volume of sample required in the analysis (100 μL), the possibility of working with replicates, the suitability of confirming the results by optical microscopy and of obtaining direct egg counts by microscopy, and the potential to develop multi-sample screening using 96 well-plates and spectrophotometer plate readers, which would allow the screening of more than 30 samples at time.

The CL detected when testing the negative HTX sediments, PMP and other reagents was expected for samples such as PMP (Fig 4), for example that is known to contain iron oxide. PMP adhere to the surface of eggs in the HTX method [38]. Such binding explains the intensified CL peak observed for the HF-eggs-PMP samples in the present study and indicates that PMP enhances CL, facilitating the distinction of positive from negative results. Stools from a healthy adult contain approximately 0.2–0.9 mg of iron [45], which is derived from exogenous sources or from mucosal bleeding [46–48]. Thus, the natural iron content of the sample is the possibly responsible for the high CL background observed in faecal samples from uninfected individuals. However, potential background induced from these sources fell well below the cut-off values for CL and did not interfere within the sensitivity and specificity of the HTX-CL. Further, such background is reduced when background samples are diluted or washed (Fig 4), a scenario that represents the samples processed by HTX, which are extensively washed during the first steps of the method.

It is noteworthy that most of the experiments reported in the literature use eggs obtained from mouse liver tissue, probably due to the difficulties associated with harvesting sufficiently large numbers of eggs from human feces. However, there are several indications that eggs in feces differ from eggs recovered from liver. Correspondingly, in the present study, higher CL intensity was exhibited by the L-eggs compared to the HF-eggs (Fig 2A and 2B). It is important to consider these differences not only in relation to tissue/fecal sources, but also in relation to human versus experimental animal sources.

In our experience with Helmintex A *lumbricoides* eggs can be found in microscopic examination of HTX samples, as those eggs are trapped by the 45 μm sieve. Because of this, we tested Ascaris eggs to determine whether they display CL. The results obtained using purified *A. lumbricoides* eggs demonstrated that they produce CL, however, lower when compared to the same number of *S*. *mansoni* eggs (Fig 3D). However, while positive CL with Ascaris eggs may present as a possible confounder in deployment of HTX-CL, this should not undermine the goal to deploy HTX-CL for schistosomiasis detection in low endemicity areas, for the following reasons: (1) HTX was designed to detect schistosome eggs and the presence of positive CL could then be confirmed by subsequent microscopic examination of the sample; (2) we only detect eggs from other parasites when they are larger than 45 μm and these are present in high number (severe infections), therefore special attention is given to the individual in terms of treatment; (3) CL positive samples are likely to be infected with a helminth and will not be likely to be egg negative; (4) we consider that these results indicate a possible application for luminol-CL beyond the schistosomiasis diagnosis. The possibility that larger numbers (50) of *A*. *lumbricoides* eggs can be associated with CL intensities similar to those produced when *S*.*mansoni* eggs are present (Fig 3D) is a challenge to be addressed in future experiments and field studies. Several procedures are under study to improve the capability of HTX to retain *S*.*mansoni* eggs while excluding *A*. *lumbricoides* and other helminths eggs: (I) a slight increase in size exclusion for the last sieving procedure, from 45 to 60 μm; (II) size exclusion sieving in a continuous tridimensional matrix: (III) differential separation using high specific gravity solutions to separately isolate different helminth eggs.

In conclusion, this innovative optimization of HTX reinforces the proposed role for the method to serve as a reference parasitological method for schistosomiasis diagnosis, and also opens up a new avenue for application of luminol-CL methods in parasitology in general. It remains to be determined whether CL can be applied to the detection of other helminths eggs with the same sensitivity and specificity. Furthermore, the feasibility of coupling the HTX-CL with immunological and molecular methods to detect and quantify *S*. *mansoni* eggs in the final sediment is yet to be assessed and could result in an even more sensitive and less laborious diagnostic method for application especially in low endemic areas, facilitating the efforts to eliminate schistosomiasis [2, 3, 4, 23]

## Supporting information

S1 Checklist STARD checklist. (DOCX)

S1 Fig STARD diagram. (DOCX)
