# Supplementary material for: A new diagnostic strategy which uses a luminol-H2O2 system to detect helminth eggs in fecal sediments processed by the Helmintex method
Source: PLoS Negl Trop Dis. 2020 Jul 30;14(7):e0008500. doi: 10.1371/journal.pntd.0008500 (PMC7437924; doi:10.1371/journal.pntd.0008500)
Supplement: S1 Checklist — (DOCX) [file pntd.0008500.s001.docx]

S1 Checklist: STARD Checklist

| **Section & Topic** | | **No** | **Item** | **Reported on page #** |
| --- | --- | --- | --- | --- |
|  | |  |  |  |
| **TITLE OR ABSTRACT** | |  |  |  |
|  | | **1** | Identification as a study of diagnostic accuracy using at least one measure of accuracy  (such as sensitivity, specificity, predictive values, or AUC) | 1 |
| **ABSTRACT** | |  |  |  |
|  | | **2** | Structured summary of study design, methods, results, and conclusions  (for specific guidance, see STARD for Abstracts) | 3 |
| **INTRODUCTION** | |  |  |  |
|  | | **3** | Scientific and clinical background, including the intended use and clinical role of the index test | 4-5 |
|  | | **4** | Study objectives and hypotheses | Page 5, Lines 104 - 112 |
| **METHODS** | |  |  |  |
| *Study design* | | **5** | Whether data collection was planned before the index test and reference standard  were performed (prospective study) or after (retrospective study) | Page 6- 14 |
| *Participants* | | **6** | Eligibility criteria | Page 10 - 13 |
|  | | **7** | On what basis potentially eligible participants were identified  (such as symptoms, results from previous tests, inclusion in registry) | Page 9, lines 194- 210 |
|  | | **8** | Where and when potentially eligible participants were identified (setting, location and dates) | *Page 6, lines 114- 126* |
|  | | **9** | Whether participants formed a consecutive, random or convenience series | Page 6- 14 |
| *Test methods* | | **10a** | Index test, in sufficient detail to allow replication | *Page 11 - 13* |
|  | | **10b** | Reference standard, in sufficient detail to allow replication | *Page 11 - 13* |
|  | | **11** | Rationale for choosing the reference standard (if alternatives exist) | No reference standard |
|  | | **12a** | Definition of and rationale for test positivity cut-offs or result categories  of the index test, distinguishing pre-specified from exploratory | *Page 16 - 18* |
|  | | **12b** | Definition of and rationale for test positivity cut-offs or result categories  of the reference standard, distinguishing pre-specified from exploratory | *Page 16 - 18* |
|  | | **13a** | Whether clinical information and reference standard results were available  to the performers/readers of the index test | Page 20 |
|  | | **13b** | Whether clinical information and index test results were available  to the assessors of the reference standard | No reference standard |
| *Analysis* | | **14** | Methods for estimating or comparing measures of diagnostic accuracy | *Page 14, lines 290- 305* |
|  | | **15** | How indeterminate index test or reference standard results were handled | *Page 14, lines 290- 305* |
|  | | **16** | How missing data on the index test and reference standard were handled | No |
|  | | **17** | Any analyses of variability in diagnostic accuracy, distinguishing pre-specified from exploratory | *Section 5*, Table 2 |
|  | | **18** | Intended sample size and how it was determined | Page 10 - 14, Resume Fig. 1 |
| **RESULTS** | |  |  | No reference standard |
| *Participants* | | **19** | Flow of participants, using a diagram | See Suplementary file 2 |
|  | | **20** | Baseline demographic and clinical characteristics of participants | Page 6, lines 121- 126 |
|  | | **21a** | Distribution of severity of disease in those with the target condition | Page 20 |
|  | | **21b** | Distribution ofalternative diagnoses in those without the target condition | No |
|  | | **22** | Time interval and any clinical interventions between index test and reference standard | No |
| *Test results* | | **23** | Cross tabulation of the index test results (or their distribution)  by the results of the reference standard | Page 18 and 19, lines 387-430 |
|  | | **24** | Estimates of diagnostic accuracy and their precision (such as 95% confidence intervals) | Page 19, lines 421-430 |
|  | | **25** | Any adverse events from performing the index test or the reference standard | **No adverse effects** |
| **DISCUSSION** | |  |  | *Section 7* |
|  | | **26** | Study limitations, including sources of potential bias, statistical uncertainty, and generalisability | *Page 21, lines 441- 563* |
|  | | **27** | Implications for practice, including the intended use and clinical role of the index test | *Page 21, lines 441- 563* |
| **OTHER INFORMATION** | |  |  |  |
|  | | **28** | Registration number and name of registry | No |
|  | | **29** | Where the full study protocol can be accessed | No |
|  | | **30** | Sources of funding and other support; role of funders | **In the electronic forms from PLoS** |
|  | |  |  |  |
|  |  |  |  |  |
|  |  |  |  |  |
|  |  |  |  |  |
|  |  |  |  |  |
