# Supplementary material for: A new diagnostic strategy which uses a luminol-H2O2 system to detect helminth eggs in fecal sediments processed by the Helmintex method
Source: PLoS Negl Trop Dis. 2020 Jul 30;14(7):e0008500. doi: 10.1371/journal.pntd.0008500 (PMC7437924; doi:10.1371/journal.pntd.0008500)
Supplement: S1 Fig — (DOCX) [file pntd.0008500.s002.docx]

S2 STARD diagram
